# Global assessment of C-reactive protein and health-related outcomes: an umbrella review of evidence from observational studies and Mendelian randomization studies

**DOI:** 10.1007/s10654-020-00681-w

**Published:** 2020-09-25

**Authors:** Georgios Markozannes, Charalampia Koutsioumpa, Sofia Cividini, Grace Monori, Konstantinos K. Tsilidis, Nikolaos Kretsavos, Evropi Theodoratou, Dipender Gill, John PA Ioannidis, Ioanna Tzoulaki

**Affiliations:** 1 Department of Hygiene and Epidemiology, University of Ioannina Medical School, 45110 Ioannina, Greece; 2 Department of Neurobiology, Harvard Medical School, Boston, MA USA; 3 BBS Program, Harvard Medical School, 220 Longwood Avenue, Boston, MA 02115 USA; 4 Department of Biostatistics, University of Liverpool, Liverpool, UK; 5 Department of Epidemiology and Biostatistics, School of Public Health, Imperial College London, London, UK; 6 Centre for Global Health, Usher Institute, University of Edinburgh, Edinburgh, UK; 7 Edinburgh Cancer Research Centre, Institute of Genetics and Molecular Medicine, University of Edinburgh, Edinburgh, UK; 8 Department of Medicine, Stanford Prevention Research Center, Stanford University School of Medicine, Stanford, CA 94305 USA; 9 Department of Health Research and Policy, Stanford University School of Medicine, Stanford, CA 94305 USA; 10 Department of Biomedical Data Sciences, Stanford University School of Medicine, Stanford, CA 94305 USA; 11 Department of Statistics, Stanford University School of Humanities and Sciences, Stanford, CA 94305 USA; 12 Meta-Research Innovation Center at Stanford (METRICS), Stanford, CA 94305 USA

**Keywords:** Umbrella review, Meta-analysis, Systematic review, C-reactive protein, CRP, Mendelian randomization, Bias

## Abstract

C-reactive protein (CRP) has been studied extensively for association with a large number of non-infectious diseases and outcomes. We aimed to evaluate the breadth and validity of associations between CRP and non-infectious, chronic health outcomes and biomarkers. We conducted an umbrella review of systematic reviews and meta-analyses and a systematic review of Mendelian randomization (MR) studies. PubMed, Scopus, and Cochrane Database of Systematic Reviews were systematically searched from inception up to March 2019. Meta-analyses of observational studies and MR studies examining associations between CRP and health outcomes were identified, excluding studies on the diagnostic value of CRP for infections. We found 113 meta-analytic comparisons of observational studies and 196 MR analyses, covering a wide range of outcomes. The overwhelming majority of the meta-analyses of observational studies reported a nominally statistically significant result (95/113, 84.1%); however, the majority of the meta-analyses displayed substantial heterogeneity (47.8%), small study effects (39.8%) or excess significance (41.6%). Only two outcomes, cardiovascular mortality and venous thromboembolism, showed convincing evidence of association with CRP levels. When examining the MR literature, we found MR studies for 53/113 outcomes examined in the observational study meta-analyses but substantial support for a causal association with CRP was not observed for any phenotype. Despite the striking amount of research on CRP, convincing evidence for associations and causal effects is remarkably limited.

**Electronic supplementary material:**

The online version of this article (10.1007/s10654-020-00681-w) contains supplementary material, which is available to authorized users.

## Introduction

C-reactive protein (CRP) is one of the most widely used biomarkers in clinical practice. First identified in 1930 [1], this acute phase reactant was initially used as a biomarker for infection [2]. The advent of high-sensitivity CRP measurement in the 1990s, alongside experimental and clinical evidence suggesting a potential role of inflammation in cardiovascular disease a few years later [3, 4], increased research interest in CRP. It has since been examined as a potential risk factor for an ever-expanding list of diseases including different cardiovascular outcomes, cancers, metabolic and skeletal diseases and autoimmune diseases [5–9]. Today, despite intensive research efforts, the role of CRP in the etiology of common diseases remains unclear.

Umbrella review is a systematic overview of systematic reviews and meta-analyses that assesses the evidence from the current literature in a field of research [10]. We aimed to systematically summarize and evaluate the breadth and validity of associations between CRP and health outcomes using the umbrella review methodology. We summarized meta-analyses of observational studies, examined the extent of phenotypic associations with CRP, and evaluated the strength of associations and bias in these identified associations. At the same time, we performed a systematic review of Mendelian randomization (MR) studies considering CRP levels as the exposure, to assess the evidence for causality stemming from this literature.

## Methods

### Data sources and searches of observational studies

We systematically searched PubMed, Scopus, and Cochrane Database of Systematic Reviews, from inception to 31 March 2019, for meta-analyses of observational studies examining the association of CRP with any health outcome (see search algorithms in Additional file 1: Appendix Table 1). All identified publications went through a three-step parallel review of title, abstract, and full text (performed by CK, GMa, SC, NK) based on predefined inclusion and exclusion criteria.

### Study selection and data extraction of observational studies

We included systematic reviews and meta-analyses of observational studies that examined associations between CRP levels and health outcomes that had identified at least three studies per outcome examined, keeping only articles that were full publications and in the English language. We excluded studies without systematic literature searches (for meta-analyses of observational studies), without quantitative synthesis of effect sizes, and studies where CRP concentrations were the outcome. Also, due to the well-known role of CRP in infectious disease diagnosis, articles which investigated infections as the outcome of interest were excluded. We also excluded meta-analyses using only cross-sectional assessments, meta-analyses of only crude (unadjusted) estimates, and associations reported as correlation coefficients. Where more than one article with overlapping outcomes was retrieved, the article with the meta-analysis of only prospective studies, the most comprehensive meta-analysis (the one including the largest number of studies), or the more recently published one was included in the final analysis (in order of preference).

Three independent investigators (CK, GMa and SC) extracted the data, which were checked by a second investigator (IT, ET) and in case of discrepancies consensus was reached. From each eligible meta-analysis, we extracted information on the first author, journal and year of publication, examined risk factors and the number of studies considered, type of metric reported (hazard ratio, risk ratio, odds ratio [OR], in order of preference), maximally adjusted effect sizes and 95% confidence intervals (CIs), number of total studies included, design of the original studies, unit of comparison, number of cases and population. When the number of cases or controls for individual studies was not reported, we abstracted them from the original studies when possible. When CRP was examined in more than one level of comparison (e.g. as a continuous biomarker and by tertiles), we extracted the data for the comparison having the largest number of component studies.

### Data synthesis and analysis of meta-analyses of observational studies

For meta-analyses of observational studies, we estimated the summary effects obtained from the random-effects method [11, 12] for which we also estimated the 95% prediction intervals to indicate the possible interval that could include the effect size of a new study examining the same association and describe the uncertainty of the summary effect size [13]. The heterogeneity between studies was assessed using the I^2^ metric, which has a range between 0 and 100%. It is calculated as the ratio of the variance between-studies over the sum of the variances between and within studies [14]. Values exceeding 50% or 75% are considered to represent large or very large heterogeneity, respectively. Small study effects were assessed with the use of the Egger’s regression asymmetry test [15]. A *P* ≤ 0.10 combined with a more conservative effect in the largest study than in random-effects meta-analysis was judged to provide evidence for small-study effects.

We further applied the excess statistical significance test, which evaluates whether there is a relative excess of formally significant findings in the published literature due to any reason (e.g., publication bias, selective reporting of outcomes or analyses) [16]. It is a Chi square-based test that assesses whether the observed number of studies with nominally significant results is larger than their expected number. We used the effect size of the largest study (smallest standard error) in each meta-analysis to calculate the power of each study using a non-central *t* distribution. Excess statistical significance was claimed at two-sided *P* ≤ 0.10 with observed > expected as previously proposed [16, 17].

### Quality assessment and evidence grading of observational studies

We classified the evidence of the associations that had *P *< 0.05 as strong, highly suggestive, suggestive, and weak based on a set of previously used criteria whose rationale has been described elsewhere in detail [10, 18–20]. In brief, these criteria try to consider the level of statistical significance, amount of evidence, consistency, and lack of signals of bias. Thus, we classified as strong evidence those associations that had significance *P *< 1×10^−6^ based on the random effects model, more than 1000 cases, the I^2^ metric was less than 50%, there was no evidence of small study effects, the prediction interval did not include the null value, and there was no evidence for excess significance bias. Associations were classified as highly suggestive when *P *< 1×10^−6^ based on the random-effects model, more than 1000 cases, and the *P* value of the largest study in the meta-analysis was < 0.05. The associations with *P *< 0.001, and more than 1000 cases were considered as suggestive. Finally, associations were considered as weak when *P *< 0.05 on the random effects model.

Some meta-analyses used estimates from studies with different study designs. Due to the inherent limitations of cross-sectional and case–control studies to examine temporal associations, we performed a sensitivity analysis by excluding cross-sectional and case–control studies.

Finally, for each association in the strong and highly suggestive category, we reassessed the evidence after examining each meta-analysis in depth by assessing the eligibility of the included studies as well as verifying the data used in the meta-analysis using AMSTAR (A MeaSurement Tool to Assess systematic Reviews) [21].

### Data sources and searches, study selection and data extraction of Mendelian randomization studies

We used the search algorithm (See Additional file 1: Appendix Table 1) to identify MR studies evaluating potential causal association between CRP levels and health outcomes, excluding infections. The titles, abstracts, and full texts of the resulting papers were examined in detail by two authors (GMa and IT), and discrepancies were resolved by consensus. From each eligible MR study, two authors (GMo and GMa) extracted data in relation to first author, journal and year of publication, the study cohort/s, sample size, number of cases (as applicable), type of data used (individual participant or summary level), the instrumental variables (single-nucleotide polymorphisms [SNPs]), the instrument selection approach, population ancestry, SNP exclusion criteria,  % variance explained by the instruments, the outcome phenotypes, the MR effect estimate and the corresponding CIs. When we observed a nominally significant association (*P *< 0.05) in the main MR analysis, we further extracted and evaluated all information on sensitivity MR analyses.

### Evidence grading of Mendelian randomization studies

We stratified MR analyses into those using instrumental variables which included only variants located in the CRP gene and those using instrumental variables with SNPs that were significantly associated with CRP levels from throughout the genome (i.e., not restricted to the *CRP* gene). The latter approach for selecting instruments is more likely to incorporate invalid instruments that have pleiotropic effects [22]. Indeed, a genome-wide association study (GWAS) of CRP has revealed a large number of genetic variants, which were not specific to CRP, but influence other inflammatory cytokines including interleukin-6 receptor (IL-6R) and interleukin 1 family member 10 (ILF10) [23]. For MR analyses restricted to variants located in the *CRP* gene, we considered MR evidence as ‘potentially supportive’ when the main analysis reported a *P *< 0.01 [20] and there was consistent evidence from sensitivity analyses; ‘limited/inconsistent evidence’ when there was 0.01 < *P *< 0.05 or *P *< 0.01 without further support from sensitivity analysis, and ‘not present’ when *P *> 0.05. For MR analyses with variants throughout the genome for CRP, we considered as ‘limited/inconsistent evidence’ when there was *P *< 0.05 and further support from sensitivity analysis, and ‘not present’ otherwise.

## Results

### CRP levels and health outcomes reported in meta-analyses of observational studies

**Fig. 1:**
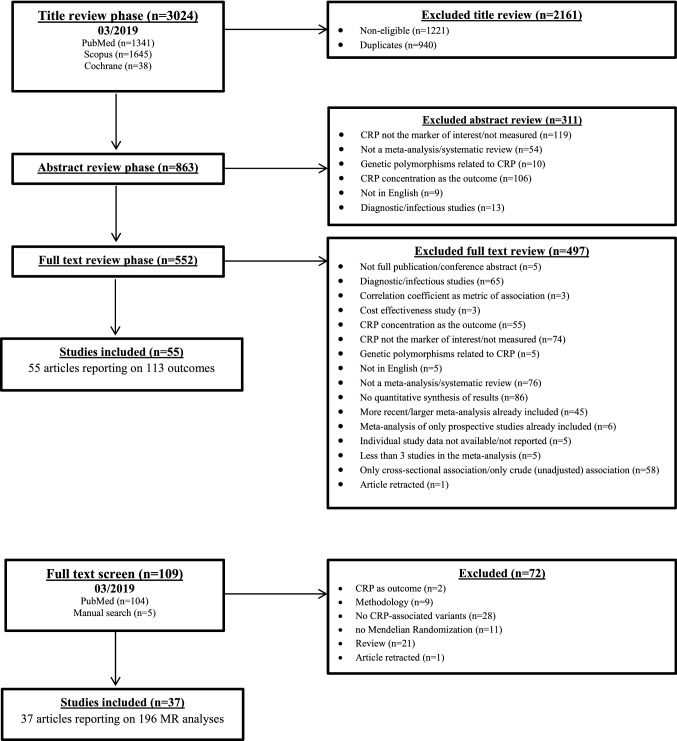
Flowchart of study selection for **a** umbrella review and **b** Mendelian Randomization review

Our literature search yielded 4100 eligible articles. Following title review, 863 articles were considered eligible (Fig. 1), and after abstract screening, 552 articles were potentially eligible for full text review. Finally, 55 studies [5, 24–77] including 113 comparisons of different outcomes were included in the umbrella review of observational studies, consisting of 952 primary estimates. To facilitate interpretation, the different outcomes were classified into the following groups: cancer-related (52 outcomes), cardiovascular-related (31 outcomes), kidney-related (7 outcomes), skeletal (6 outcomes), neurological (3 outcomes), pregnancy-related (2 outcomes), respiratory-related (2 outcomes), and other (10 outcomes).

The majority of the primary studies were cohorts (N = 823; 86.5%, of which 497 were prospective, 264 retrospective, and 62 of unclear design), followed by case–control studies (N = 115; 12.1%). Other study designs consisted of cross-sectional studies (N = 6; 0.6%), case-cohorts (N = 7; 0.7%), and one case-crossover study (0.1%).

Ninety-five out of 113 associations (84.1%) presented a statistically significant effect at *P *< 0.05 under the random-effects model, 67 remained significant at *P* < 0.001, whereas 34 associations had a statistically significant effect at *P *< 1×10^−6^ (Table 1). However, only 24 (21.2%) associations had a 95% prediction interval that excluded the null. The largest study was statistically significant in 71 of the 113 comparisons (62.8%) and was more conservative than the meta-analysis estimate in 87 of 113 comparisons (77%) (Table 1). Twenty-three associations (20.4%) presented very large between-study heterogeneity (I^2^ > 75%), and 31 (27.4%) associations had large heterogeneity estimates (I^2^ > 50% and I^2^ < 75%). In 45 (39.8%) of the 113 associations the Egger’s test was statistically significant (*P *< 0.1) and the random effects estimate was inflated compared to the largest study (Table 1). Forty-seven associations (41.6%) showed evidence of excess significance, meaning that the number of observed studies with statistically significant results exceeded the number of expected ones (Table 1).

**Table 1:** Health outcomes and assessment of evidence in meta-analyses of observational studies

References	Contrast	Population	Outcome	Meta-analysis metric	N Studies	N cases/N population	Random effects (95% CI)^a^	Random effects *P*	Largest study (95% CI)^b^	Prediction interval	I^2^	Egger’s *P*^c^	Excess Significance		Evidence Grade
													O/E	*P*^d^	
*Cancer*-*related outcomes*															
Zheng et al. [69]	High vs Low	Hepatocellular carcinoma	Overall survival	HR	11	1071/1885	2.15 (1.76, 2.63)	1.0E − 13	1.80 (1.30, 2.30)	1.36, 3.39	27 (0, 64)	0.171	9/4.49	0.010	Highly suggestive
Zeng et al. [67]	High vs Low	General population (women)	Ovarian cancer	RR	7	2011/33288	1.91 (1.51, 2.41)	5.0E − 08	1.67 (1.03, 2.70)	1.41, 2.59	0 (0, 58)	0.015^d^	4/6.14	NP	Highly suggestive
Liao et al. [48]	High vs Low	Non-small cell lung carcinoma	Overall survival	HR	14	1342/2491	1.63 (1.36, 1.94)	1.0E − 07	1.03 (1.00, 1.06)	0.88, 3.01	90 (85, 93)	0.002^d^	11/2.61	2.0E − 06	Suggestive
Guo et al. [32]	per unit lnCRP	Breast cancer	Overall survival	HR	13	3180/15112	1.28 (1.13, 1.44)	5.9E − 05	1.03 (1.00, 1.06)	0.89, 1.83	77 (58, 85)	0.004^d^	6/0.71	3.3E − 05	Suggestive
Li et al. [20]	High vs Low	General population	Cancer mortality	RR	8	4748/55720	1.26 (1.11, 1.42)	1.9E − 04	1.28 (1.11, 1.48)	1.00, 1.58	17 (0, 63)	0.505	3/5.33	NP	Suggestive
Guo et al. [32]	per unit lnCRP	General population	Lung cancer	HR	7	1045/127867	1.34 (1.15, 1.57)	2.3E − 04	1.51 (1.21, 1.88)	0.89, 2.03	45 (0, 75)	0.600	4/3.80	1.000	Suggestive
Guo et al. [33]	per unit lnCRP	Breast cancer	Cancer-specific survival	HR	7	1320/12932	1.38 (1.15, 1.66)	6.5E − 04	1.16 (1.02, 1.32)	0.86, 2.20	51 (0, 77)	0.095^d^	4/1.22	0.021	Suggestive
Wang et al. [59]	High vs Low	Renal cell carcinoma, receiving tyrosine kinase inhibitors	Overall survival	HR	8	490/1158	2.83 (2.26, 3.56)	2.5E − 19	3.17 (2.20, 4.68)	1.90, 4.22	12 (0, 61)	0.226	6/7.11	NP	Weak
Yu et al. [66]	High vs Low	Gastric cancer	Overall survival	HR	12	771/2597	1.77 (1.56, 2.00)	3.9E − 19	1.54 (1.25, 1.92)	1.38, 2.27	19 (0, 59)	0.207	9/4.54	0.013	Weak
Hu et al. [38]	High vs Low	Metastatic renal cell carcinoma	Overall survival	HR	5	487/729	2.56 (2.05, 3.19)	1.2E − 16	2.10 (1.50, 3.00)	1.78, 3.67	0 (0, 64)	0.795	5/3.08	0.164	Weak
Fang et al. [30]	High vs Low	Nasopharyngeal carcinoma	Overall survival	HR	5	439/3691	1.84 (1.57, 2.17)	1.9E − 13	1.82 (1.47, 2.25)	1.41, 2.40	0 (0, 64)	0.168	5/3.11	0.164	Weak
Dai et al. [29]	High vs Low	Renal cell carcinoma	Overall survival	HR	12	865/2305	2.51 (1.93, 3.26)	4.7E − 12	1.20 (1.15, 1.26)	1.06, 5.95	93 (90, 95)	0.002^d^	11/3.85	3.1E − 05	Weak
Fang et al. [30]	High vs Low	Nasopharyngeal carcinoma	Distant metastasis-free survival	HR	3	449/3513	1.81 (1.52, 2.14)	1.0E − 11	1.71 (1.38, 2.13)	0.60, 5.47	0 (0, 73)	0.061^d^	3/2.35	1.000	Weak
Zheng et al. [69]	High vs Low	Hepatocellular carcinoma	TNM stage	HR	3	185/689	3.23 (2.29, 4.56)	2.7E − 11	3.29 (2.22, 4.88)	0.34, 30.25	0 (0, 73)	0.808	2/2.39	NP	Weak
Hu et al. [38]	High vs Low	Localised renal cell carcinoma	Progression-free survival	HR	4	233/881	3.27 (2.25, 4.77)	6.9E − 10	3.26 (1.79, 6.53)	1.43, 7.49	0 (0, 68)	0.721	4/3.58	1.000	Weak
Woo et al. [61]	High vs Low	Colorectal cancer	Cancer-specific survival	HR	3	126/579	4.37 (2.63, 7.26)	1.3E − 08	4.90 (2.33, 10.31)	0.16, 117.79	0 (0, 73)	0.594	3/2.99	1.000	Weak
Dai et al. [29]	High vs Low	Renal cell carcinoma	Cancer-specific survival	HR	12	783/2843	3.52 (2.18, 5.69)	2.7E − 07	1.23 (1.17, 1.30)	0.63, 19.74	92 (88, 94)	1.4E − 04^d^	12/3.19	1.3E − 07	Weak
Dai et al. [29]	High vs Low	Upper urinary tract and bladder cancer	Overall survival	HR	3	278/408	1.63 (1.33, 1.99)	2.7E − 06	1.56 (1.18, 2.06)	0.44, 6.08	0 (0, 73)	0.043^d^	3/0.60	0.008	Weak
Luo et al. [50]	High vs Low	Urothelial bladder cancer	Cancer-specific survival	HR	4	373/1495	1.64 (1.32, 2.03)	7.3E − 06	1.96 (1.42, 2.69)	0.86, 3.12	21 (0, 74)	0.937	2/3.21	NP	Weak
Dai et al. [29]	High vs Low	Upper urinary tract and bladder cancer	Cancer-specific survival	HR	8	411/1384	1.81 (1.39, 2.36)	1.3E − 05	1.20 (1.10, 1.30)	0.83, 3.97	73 (31, 85)	3.0E − 04^d^	8/0.72	4.5E − 09	Weak
Hu et al. [38]	High vs Low	Localised renal cell carcinoma	Cancer-specific survival	HR	3	102/759	3.40 (1.95, 5.92)	1.6E − 05	3.87 (1.70, 8.82)	0.09, 124.54	0 (0, 73)	0.571	2/2.69	NP	Weak
Liu et al. [40]	per unit lnCRP	Prostate cancer	Progression-free survival	HR	3	54/316	1.50 (1.25, 1.81)	1.9E − 05	1.44 (1.17, 1.77)	0.45, 5.07	0 (0, 73)	0.568	2/2.18	NP	Weak
Woo et al. [61]	High vs Low	Colorectal cancer	Overall survival	HR	4	184/778	2.04 (1.45, 2.86)	4.0E − 05	1.88 (1.10, 3.20)	0.97, 4.29	0 (0, 68)	0.290	3/1.99	0.372	Weak
Zheng [69]	High vs Low	Hepatocellular carcinoma	Tumor vascular invasion	HR	5	256/915	3.05 (1.78, 5.23)	4.7E − 05	4.11 (2.58, 6.53)	0.66, 14.22	44 (0, 78)	0.494	3/4.32	NP	Weak
Wang et al. [114]	High vs Low	Renal cell carcinoma, receiving tyrosine kinase inhibitors	Progression-free survival	HR	4	81/449	2.35 (1.53, 3.63)	1.0E − 04	2.48 (1.74, 3.59)	0.60, 9.18	23 (0, 75)	0.999	3/1.91	0.355	Weak
Liu et al. [49]	High vs Low	Prostate cancer	Cancer-specific survival	HR	4	162/822	1.91 (1.36, 2.69)	2.2E − 04	1.48 (0.83, 2.66)	0.89, 4.09	1 (0, 68)	0.020^d^	2/1.75	1.000	Weak
Rocha et al. [54]	High vs Low	Metastatic prostate cancer	Overall survival	HR	6	432/659	1.42 (1.18, 1.72)	2.8E − 04	1.11 (1.02, 1.20)	0.81, 2.51	72 (8, 86)	0.006^d^	5/0.35	3.6E − 06	Weak
Dai et al. [29]	High vs Low	Renal cell carcinoma	Recurrence-free survival	HR	8	189/1485	3.09 (1.66, 5.74)	3.8E − 04	1.23 (1.14, 1.33)	0.39, 24.50	89 (82, 93)	0.012^d^	7/2.52	0.002	Weak
Huang et al. [39]	High vs Low	Esophageal cancer	Overall survival	HR	8	683/1329	2.00 (1.36, 2.94)	4.0E − 04	1.18 (1.03, 1.36)	0.59, 6.83	81 (60, 89)	0.027^d^	6/0.64	6.1E − 06	Weak
Zheng et al. [69]	High vs Low	Hepatocellular carcinoma	Recurrence-free survival	HR	3	245/445	2.66 (1.54, 4.58)	4.3E − 04	3.05 (1.68, 5.52)	0.02, 410.95	34 (0, 81)	0.799	2/2.44	NP	Weak
Zhou et al. [70]	per unit lnCRP	General population	Colorectal cancer	RR	18	4779/152418	1.12 (1.05, 1.21)	1.3E − 03	1.06 (0.99, 1.13)	0.90, 1.41	52 (4, 71)	0.069^d^	6/1.28	0.001	Weak
Zhou et al. [70]	per unit lnCRP	General population	Colon cancer	RR	13	9715/153763	1.12 (1.05, 1.21)	1.4E − 03	1.00 (0.92, 1.07)	0.94, 1.34	38 (0, 66)	0.052^d^	4/0.65	0.003	Weak
Liu et al. [49]	High vs Low	Prostate cancer	Overall survival	HR	4	273/471	1.38 (1.13, 1.68)	0.002	1.11 (1.02, 1.20)	0.57, 3.31	82 (35, 91)	0.038^d^	4/0.24	1.2E − 05	Weak
Dai et al. [29]	High vs Low	Upper urinary tract and bladder cancer	Recurrence-free survival	HR	3	266/727	1.62 (1.20, 2.19)	0.002	1.45 (1.06, 1.99)	0.10, 27.49	36 (0, 81)	0.133	3/0.88	0.025	Weak
Guo et al. [33]	per unit lnCRP	General population	Any cancer	HR	11	11459/194796	1.11 (1.04, 1.18)	0.002	1.00 (0.94, 1.07)	0.89, 1.37	70 (34, 82)	0.717	5/0.55	1.1E − 04	Weak
Hu et al. [38]	High vs Low	Clear cell renal cell carcinoma	Cancer-specific survival	HR	3	269/522	2.98 (1.48, 6.00)	0.002	2.64 (1.04, 6.70)	0.01, 1243.25	25 (0, 79)	0.619	2/2.55	NP	Weak
Zheng et al. [69]	High vs Low	Hepatocellular carcinoma	Tumor number	HR	5	448/935	2.36 (1.36, 4.10)	0.002	1.81 (1.23, 2.66)	0.40, 14.08	62 (0, 84)	0.568	4/2.59	0.377	Weak
Leuzzi et al. [44]	High vs Low	Early stage non-small cell lung carcinoma	Mortality	HR	10	2106/3165	1.42 (1.11, 1.81)	0.005	1.06 (0.95, 1.18)	0.64, 3.16	81 (63, 88)	0.162	8/0.58	5.0E − 09	Weak
Wang et al. [58]	High vs Low	General population (women)	Breast cancer	RR	11	5371/69157	1.26 (1.06, 1.49)	0.007	0.89 (0.76, 1.06)	0.79, 2.02	50 (0, 73)	0.006^d^	2/2.15	NP	Weak
Guo et al. [32]	per unit lnCRP	Breast cancer	Disease-free survival	HR	9	1790/8350	1.18 (1.04, 1.34)	0.009	1.03 (1.00, 1.07)	0.83, 1.69	76 (47, 86)	0.080^d^	3/0.48	0.010	Weak
Godos et al. [31]	High vs Low	Patients who underwent sigmoidoscopy/colonoscopy	Advanced adenoma	OR	4	1092/2330	1.59 (1.09, 2.32)	0.016	1.10 (0.76, 1.59)	0.41, 6.14	44 (0, 80)	0.431	2/0.39	0.050	Weak
Qin et al. [73]	High vs Low	Diffuse large B-cell lymphoma patients	Overall survival	HR	11	579/2681	2.67 (1.95, 3.64)	6.7E − 10	1.51 (1.04, 2.20)	1.06, 6.67	60 (2, 78)	0.029^d^	11/5.76	0.001	Weak
Qin et al. [112]	High vs Low	Diffuse large B-cell lymphoma patients	Progression-free survival	HR	5	353/1269	2.19 (1.68, 2.86)	7.4E − 09	1.91 (1.28, 2.85)	1.22, 3.92	16 (0, 70)	0.961	4/3.78	1.000	Weak
Li et al. [20]	High vs Low	Patients with bone neoplasms	Overall survival	HR	5	315/816	1.87 (1.28, 2.75)	0.001	1.40 (1.00, 1.80)	0.54, 6.45	62 (0, 84)	0.473	4/0.97	0.006	Weak
Chen et al. [77]	High vs Low	Pancreatic cancer patients	Overall survival	HR	5	266/551	2.28 (1.38, 3.79)	0.001	1.36 (0.99, 1.88)	0.43, 12.06	71 (0, 87)	0.009^d^	3/1.29	0.112	Weak
Godos et al. [31]	High vs Low	Patients who underwent sigmoidoscopy/colonoscopy	Colorectal adenoma (total)	OR	12	3350/8308	1.23 (0.98, 1.54)	0.077	1.10 (0.76, 1.59)	0.61, 2.46	54 (0, 75)	0.322	3/1.23	0.117	NS
Guo et al. [33]	per unit lnCRP	General population (men)	Prostate cancer	HR	5	1586/48450	1.07 (0.98, 1.17)	0.156	1.12 (0.97, 1.30)	0.92, 1.24	0 (0, 64)	0.482	0/0.94	NP	NS
Zheng et al. [69]	High vs Low	Hepatocellular carcinoma	Tumor differentiation	HR	3	46/364	1.58 (0.74, 3.40)	0.237	2.26 (0.85, 6.01)	0.01, 223.37	0 (0, 73)	0.324	0/1.01	NP	NS
Zhang et al. [68]	High vs Low	General population	Colorectal adenoma	RR	11	6303/14925	1.11 (0.89, 1.38)	0.347	1.32 (1.45, 0.57)	0.58, 2.13	64 (2, 80)	0.574	4/6.52	NP	NS
Hu et al. [38]	High vs Low	Clear cell renal cell carcinoma	Overall survival	HR	3	220/607	1.32 (0.66, 2.65)	0.426	1.43 (0.86, 2.39)	0.00, 1537.81	48 (0, 84)	0.699	0/0.68	NP	NS
Zhou 2014 [70]	per unit lnCRP	General population	Rectal cancer	RR	12	1170/48209	1.03 (0.90, 1.17)	0.705	0.99 (0.88, 1.10)	0.72, 1.46	43 (0, 69)	0.923	3/0.60	0.020	NS
Godos et al. [31]	High vs Low	Patients who underwent sigmoidoscopy/colonoscopy	Non-advanced adenoma	OR	5	536/1625	1.06 (0.57, 1.98)	0.843	0.77 (0.46, 1.29)	0.12, 9.44	77 (20, 89)	0.972	3/1.13	0.080	NS
*Cardiovascular*-*related outcomes*															
Li et al. [47]	High vs Low	General population	CVD mortality	RR	6	1612/35727	2.05 (1.64, 2.57)	3.6E − 10	1.49 (1.00, 2.21)	1.34, 3.13	13 (0, 66)	0.115	5/5.05	NP	Strong
Kunutsot et al. [43]	per 1 SD lnCRP	General population	Venous thromboembolism	HR	9	2225/81625	1.14 (1.08, 1.19)	2.9E − 07	1.18 (1.06, 1.32)	1.07, 1.21	0 (0, 54)	0.743	3/2.48	0.714	Strong
Heming et al. [35]	High vs Low	Stable CAD	Mortality or CVD	RR	53	5244/50519	1.94 (1.71, 2.20)	5.2E − 25	1.14 (1.06, 1.23)	0.97, 3.88	77 (70, 82)	4.8E − 11^d^	38/7.21	9.1E − 22	Highly suggestive
ERFC [41]	per 1 SD lnCRP	General population	CHD	HR	31	5373/111899	1.38 (1.27, 1.49)	6.6E − 16	1.27 (1.11, 1.44)	1.09, 1.73	26 (0, 52)	0.724	16/10.59	0.056	Highly suggestive
He et al. [78]	High vs Low	ACS/unstable CHD/angina	Mortality or CVD (long-term)	RR	11	1276/9011	2.18 (1.78, 2.68)	8.6E − 14	1.70 (1.30, 2.60)	1.21, 3.93	50 (0, 73)	0.024^d^	9/8.95	1.000	Highly suggestive
Bibek et al. [26]	High vs Low	Patients undergoing PCI	MACE	RR	33	4120/34367	1.96 (1.65, 2.34)	2.8E − 14	1.10 (1.00, 1.20)	0.86, 4.50	84 (79, 88)	1.5E − 05^d^	24/2.72	1.7E − 19	Suggestive
Bibek et al. [26]	High vs Low	Patients undergoing PCI	Mortality	RR	26	1358/33068	3.00 (2.18, 4.12)	1.4E − 11	1.08 (0.93, 1.24)	0.84, 10.69	78 (68, 84)	1.4E − 04^d^	15/1.57	2.1E − 12	Suggestive
Xu et al. [63]	per 1 mg/L CRP	General population	Ischemic stroke	RR	10	3071/125260	1.15 (1.09, 1.22)	1.2E − 06	1.09 (1.04, 1.14)	1.01, 1.30	37 (0, 69)	0.006^d^	6/1.18	3.8E − 04	Suggestive
Saito et al. [55]	High vs Low	East Asians	CHD	RR	3	1319/310964	1.76 (1.29, 2.40)	3.4E − 04	1.39 (1.04, 1.86)	0.08, 40.61	49 (0, 84)	0.547	3/2.38	1.000	Suggestive
Correia et al. [28]	High vs Low	ACS	Mortality or CVD (long-term)	OR	6	424/3270	4.58 (2.78, 7.53)	2.1E − 09	2.80 (1.81, 4.32)	1.00, 20.86	69 (0, 85)	0.016^d^	6/5.49	1.000	Weak
Yo et al. [65]	High vs Low	AF	AF recurrence	OR	9	333/632	4.05 (2.51, 6.54)	9.3E − 09	1.60 (1.00, 2.50)	0.95, 17.34	66 (12, 82)	3.6E − 04^d^	9/1.61	1.9E − 07	Weak
Bibek et al. [26]	High vs Low	Patients undergoing PCI	MI	RR	24	974/23271	1.80 (1.47, 2.21)	1.0E − 08	1.42 (1.14, 1.76)	1.00, 3.25	42 (0, 63)	0.003^d^	7/4.71	0.299	Weak
Singh et al. [56]	High vs Low	Peripheral artery disease	Major CVD	HR	4	194/752	2.26 (1.65, 3.09)	3.5E − 07	1.89 (1.18, 3.02)	1.14, 4.49	0 (0, 68)	0.185	4/2.08	0.126	Weak
Mincu et al. [52]	High vs Low	Patients with STEMI	All-cause mortality	RR	6	142/2721	2.68 (1.78, 4.04)	2.2E − 06	2.62 (1.94, 3.50)	0.96, 7.53	49 (0, 78)	0.136	4/3.04	0.688	Weak
Mincu et al. [52]	High vs Low	Patients with STEMI	Recurrent MI	RR	4	28/1480	3.51 (1.90, 6.48)	5.8E − 05	2.84 (1.27, 6.35)	0.92, 13.47	0 (0, 68)	0.422	2/1.23	0.591	Weak
Singh et al. [56]	per unit lnCRP	Peripheral artery disease	Major CVD	HR	5	179/1184	1.38 (1.16, 1.63)	2.1E − 04	1.47 (1.13, 1.98)	0.97, 1.95	12 (0, 68)	0.449	2/1.03	0.276	Weak
Bibek et al. [26]	High vs Low	Patients undergoing PCI	Coronary revascularization	RR	21	2115/21694	1.31 (1.11, 1.56)	0.002	0.91 (0.81, 1.02)	0.71, 2.43	69 (47, 79)	0.001^d^	5/1.63	0.020	Weak
Correia et al. [28]	High vs Low	ACS	Mortality or CVD (short-term)	OR	12	1203/13256	1.65 (1.20, 2.27)	0.002	1.45 (1.20, 1.74)	0.68, 3.98	62 (14, 78)	0.546	6/5.29	0.775	Weak
Padayachee et al. [53]	High vs Low	Vascular surgery	MACE	OR	3	67/386	2.74 (1.36, 5.51)	0.005	2.55 (1.12, 5.83)	0.03, 252.05	0 (0, 73)	0.916	1/1.49	NP	Weak
Saito et al. [55]	High vs Low	East Asians	Stroke	RR	6	2292/91852	1.40 (1.10, 1.77)	0.006	0.93 (0.64, 1.35)	0.78, 2.49	33 (0, 73)	0.116	2/0.73	0.158	Weak
Saito et al. [55]	High vs Low	East Asians	Ischemic stroke	RR	4	1226/85331	1.40 (1.08, 1.81)	0.010	1.19 (0.82, 1.73)	0.80, 2.46	0 (0, 68)	0.018^d^	0/1.46	NP	Weak
Mincu et al. [52]	High vs Low	Patients with STEMI	In-hospital target revascularization	RR	3	13/1222	3.17 (1.30, 7.72)	0.011	4.53 (1.44, 14.23)	0.00, 7582.37	27 (0, 79)	0.234	2/1.19	0.567	Weak
Bibek et al. 2014 [26]	High vs Low	Patients undergoing PCI	Restenosis	RR	9	511/2765	1.45 (1.07, 1.96)	0.016	1.10 (0.83, 1.45)	0.63, 3.37	59 (0, 79)	0.431	4/0.58	0.002	Weak
Padayachee et al. [53]	High vs Low	Vascular surgery	Cardiac death	OR	4	34/477	4.15 (1.18, 14.52)	0.026	5.38 (0.62, 46.50)	0.26, 64.96	0 (0, 68)	0.552	1/2.50	NP	Weak
Barron et al. [25]	per 1 SD CRP	Adults (mean age: 50–75)	CVD mortality	HR	3	569/7269	1.31 (1.02, 1.69)	0.033	1.28 (1.14, 1.44)	0.07, 23.53	81 (0, 92)	0.582	2/1.28	0.579	Weak
Padayachee et al. [53]	High vs Low	Vascular surgery	All-cause mortality (long-term)	OR	4	53/530	2.19 (1.02, 4.67)	0.043	3.43 (1.15, 10.28)	0.40, 11.83	1 (0, 68)	0.889	1/2.16	NP	Weak
Yu et al. [76]	High vs Low	Patients with acute ischemic stroke	All-cause mortality	HR	6	663/3035	2.45 (1.47, 4.06)	5.4E − 04	2.00 (1.70, 1.30)	0.48, 12.52	29 (0, 76)	0.912	5/5.07	NP	Weak
Saito et al. [55]	High vs Low	East Asians	CHD	RR	4	625/74626	1.75 (0.96, 3.19)	0.068	1.13 (0.70, 1.82)	0.13, 22.80	72 (0, 88)	0.159	2/0.53	0.089	NS
Barron et al. [25]	per 1 SD CRP	Adults (mean age: 50–75)	CHD mortality	HR	3	333/7269	1.20 (0.93, 1.56)	0.160	1.27 (1.09, 1.48)	0.07, 21.28	71 (0, 89)	0.760	2/0.81	0.181	NS
Padayachee et al. [53]	High vs Low	Vascular surgery	MI (nonfatal)	OR	3	36/386	1.37 (0.62, 3.00)	0.436	1.24 (0.52, 2.97)	0.01, 222.04	0 (0, 73)	0.319	0/0.20	NP	NS
Saito et al. [55]	High vs Low	East Asians	Hemorrhagic stroke	RR	4	863/85331	1.04 (0.66, 1.65)	0.850	0.70 (0.46, 1.07)	0.21, 5.16	39 (0, 79)	0.061	0/2.62	NP	NS
*Kidney*-*related outcomes*															
Li et al. [46]	High vs Low	Chronic kidney disease	All-cause mortality	HR	17	2327/9022	1.21 (1.14, 1.29)	5.6E − 10	1.02 (1.01, 1.03)	1.01, 1.46	89 (84, 92)	3.9E − 05^d^	14/1.82	1.3E − 11	Highly suggestive
Li et al. [46]	High vs Low	Chronic kidney disease	CVD mortality	HR	14	7685.966/14498	1.19 (1.10, 1.28)	2.3E − 05	1.02 (1.01, 1.03)	0.95, 1.49	76 (57, 84)	1.1E − 04^d^	7/0.75	3.1E − 06	Suggestive
Herselman et al. [36]	per 1 mg/L CRP	Dialysis	All-cause mortality	HR	9	503/1608	1.03 (1.02, 1.05)	1.9E − 04	1.02 (1.01, 1.03)	0.99, 1.08	74 (40, 85)	0.001^d^	8/0.45	3.5E − 10	Weak
Avram et al. [24]	High vs Low	Peritoneal dialysis	All-cause mortality	HR	15	619/3333	1.04 (1.02, 1.06)	3.0E − 04	1.02 (1.01, 1.03)	0.98, 1.10	80 (67, 87)	1.5E − 04^d^	11/0.76	6.0E − 12	Weak
Herselman et al. [36]	per 1 mg/L CRP	Dialysis	CVD mortality	HR	4	137/1047	1.06 (0.98, 1.15)	0.133	1.00 (0.98, 1.02)	0.77, 1.46	86 (59, 93)	0.075^d^	2/0.20	0.014	NS
Chan et al. [27]	High vs Low	Children with HSP	HSP nephritis	OR	5	380/955	1.33 (0.78, 2.28)	0.298	1.20 (0.78, 1.83)	0.26, 6.91	56 (0, 82)	0.907	1/0.52	0.420	NS
Shen (2016)	High vs Low	Peritoneal dialysis	CVD mortality	HR	5	134/832	1.69 (0.50, 5.74)	0.403	1.03 (1.01, 1.05)	0.02, 146.16	91 (83, 95)	0.794	4/0.25	3.1E − 05	NS
*Skeletal*-*related outcomes*															
Maneiro et al. [51]	High vs Low	AS on anti-TNF	BASDAI50	OR	6	1384/2570	2.14 (1.71, 2.68)	2.5E − 11	1.94 (1.53, 2.45)	1.32, 3.48	22 (0, 69)	0.015^d^	6/3.99	0.188	Highly suggestive
Wu et al. [62]	High vs Low	General population	Fracture	RR	6	2421/14382	2.14 (1.51, 3.05)	2.2E − 05	1.78 (1.27, 2.46)	0.75, 6.11	62 (0, 82)	0.047^d^	5/5.09	NP	Suggestive
Maneiro et al. [51]	High vs Low	AS on anti-TNF	ASAS20	OR	6	865/1262	2.53 (2.00, 3.21)	1.7E − 14	2.18 (1.34, 3.53)	1.81, 3.54	0 (0, 61)	0.508	5/4.58	1.000	Weak
Maneiro et al. [51]	High vs Low	AS on anti-TNF	ASAS40	OR	3	758/1524	2.03 (1.49, 2.76)	7.0E − 06	2.02 (1.60, 2.55)	0.12, 33.85	28 (0, 80)	0.559	3/2.11	0.560	Weak
Maneiro et al. [51]	High vs Low	AS on anti-TNF	BASDAI	OR	5	940/1617	1.04 (1.01, 1.08)	0.004	1.02 (1.01, 1.04)	0.94, 1.16	86 (64, 92)	0.092^d^	4/0.26	3.3E − 05	Weak
Jin et al. [40]	High vs Low	Osteoarthritis	Disease progression	OR	4	2469/10619	0.97 (0.71, 1.33)	0.855	1.12 (0.81, 1.54)	0.28, 3.40	57 (0, 84)	0.598	1/1.08	NP	NS
*Neurological*-*related outcomes*															
Koyama et al. [42]	High vs Low	General population	Dementia	HR	5	746/4392	1.45 (1.10, 1.91)	0.008	1.21 (0.85, 1.73)	0.68, 3.11	39 (0, 76)	0.358	1/1.06	NP	Weak
Koyama et al. [42]	High vs Low	General population	Alzheimer’s disease	HR	7	565/5401	1.21 (1.03, 1.42)	0.021	1.23 (1.00, 1.52)	0.98, 1.49	0 (0, 58)	0.913	0/1.12	NP	Weak
Yang et al. [64]	High vs Low	Non-demented adults	Cognitive decline	RR	4	1001/5170	1.29 (0.95, 1.75)	0.101	1.24 (0.96, 1.63)	0.49, 3.39	25 (0, 75)	0.939	1/1.46	NP	NS
*Respiratory*-*related outcomes*															
Leuzzi et al. [45]	High vs Low	COPD	Mortality (late)	HR	15	2287/11728	1.53 (1.32, 1.77)	1.5E − 08	1.48 (1.28, 1.71)	0.93, 2.52	69 (40, 80)	0.010^d^	12/7.49	0.021	Highly suggestive
Leuzzi et al. [45]	High vs Low	COPD	Mortality (early)	RR	11	802/6688	1.15 (0.93, 1.42)	0.183	1.22 (1.11, 1.34)	0.60, 2.21	87 (78, 91)	0.517	8/1.44	9.6E − 06	NS
*Pregnancy*-*related outcomes*															
Wei et al. [60]	High vs Low (plasma CRP)	Pregnant women	Spontaneous preterm birth	OR	5	934/3543	1.61 (1.22, 2.11)	6.6E − 04	1.17 (0.84, 1.63)	0.81, 3.18	27 (0, 73)	0.040^d^	3/0.83	0.035	Weak
Wei et al. [60]	High vs Low (amniotic fluid CRP)	Pregnant women	Spontaneous preterm birth	OR	3	165/647	8.75 (1.86, 41.12)	0.006	2.80 (0.99, 7.94)	0.00, 3.11E + 08	68 (0, 89)	0.068^d^	2/2.69	NP	Weak
*Other outcomes*															
Li et al. [47]	High vs Low	General population	All-cause mortality	RR	14	9285/71016	1.75 (1.55, 1.98)	8.3E − 19	1.49 (1.24, 1.78)	1.16, 2.64	60 (16, 77)	0.192	12/12.97	NP	Highly suggestive
Wang et al. [5]	per unit lnCRP	General population	Type 2 diabetes	RR	22	5836/40435	1.26 (1.16, 1.37)	5.8E − 08	1.17 (1.06, 1.29)	0.92, 1.71	64 (38, 76)	0.204	11/4.59	0.002	Highly suggestive
Hong et al. [37]	High vs Low	Adults ≥ 40 yeas	Age-related macular degeneration	OR	11	3232/41690	1.69 (1.28, 2.23)	2.2E − 04	1.24 (0.87, 1.78)	0.78, 3.63	51 (0, 74)	0.004^d^	4/3.56	0.755	Suggestive
Wu et al. [75]	High vs Low	Patients receiving allogeneic stem cell transplant	Overall survival	HR	14	1275/3216	1.63 (1.34, 1.98)	8.8E − 07	0.96 (0.91, 1.13)	0.85, 3.12	77 (59, 85)	3.7E − 04^d^	8/2.64	0.002	Suggestive
Tian et al. [74]	High vs Low	Type 2 diabetic patients	All-cause mortality	RR	6	1121/9843	2.03 (1.49, 2.75)	6.5E − 06	1.77 (1.29, 2.42)	0.82, 5.00	60 (0, 82)	0.167	4/5.27	NP	Suggestive
Jayedi et al. [71]	High vs Low	General population	Hypertension	RR	12	18877/137918	1.26 (1.13, 1.39)	1.5E − 05	1.09 (1.03, 1.16)	0.94, 1.68	65 (23, 80)	0.153	7/3.78	0.060	Suggestive
Tian et al. [80]	High vs Low	Type 2 diabetic patients	Cardiovascular mortality	RR	6	1451/21148	1.74 (1.35, 2.23)	1.7E − 05	2.09 (1.57, 2.77)	0.98, 3.08	28 (0, 71)	0.944	3/5.35	NP	Suggestive
Wu et al. [75]	High vs Low	Patients receiving allogeneic stem cell transplant	non-relapse mortality	HR	14	513/3128	2.06 (1.62, 2.62)	4.4E − 09	1.50 (1.24, 1.82)	1.03, 4.12	52 (0, 72)	0.007^d^	8/4.87	0.094	Weak
Wu et al. [75]	High vs Low	Patients receiving allogeneic stem cell transplant	acute graft versus host disease	HR	7	104/1133	1.35 (1.07, 1.71)	0.013	1.00 (0.98, 1.01)	0.71, 2.57	77 (40, 87)	0.002^d^	4/2.25	0.222	Weak
Soysal et al. [57]	High vs Low	Elderly	Frailty	OR	3	1045/2939	1.06 (0.78, 1.44)	0.694	1.05 (0.72, 1.54)	0.15, 7.68	0 (0, 73)	0.678	0/0.20	NP	NS

All statistical tests were two-sided

*ACS* Acute coronary syndrome; *AF* Atrial fibrillation; *anti*-*TNF* anti-tumor necrosis factor; *AS* Ankylosing spondylitis; *ASAS* Assessment in Ankylosing Spondylitis response criteria; *BASDAI* Bath Ankylosing Spondylitis Disease Activity Index; *CAD* Coronary artery disease; *CHD* Coronary heart disease; *CI* confidence interval; *COPD* Chronic obstructive pulmonary disease; *CRP* C-reactive protein; *CVD* Cardiovascular disease; *HR* Hazard ratio; *HSP* Henoch-Schönlein purpura; *MACE* Major Adverse Cardiac Events; *MI* Myocardial infarction; *NP* Not pertinent; *NS* Not significant; *OR* Odds ratio; *RR* Relative risk; *STEMI* ST-elevation myocardial infarction

^a^Random-effects refers to summary relative risk (95% CI) using the meta-analysis random-effects model

^b^Largest study (smallest standard error)

^c^*P*-value from the Egger’s regression asymmetry test

^d^Denotes doth a *P*-value < 0.1 and that the largest study is more conservative that the summary random effects estimate

^e^*P*-value of the excess statistical significance test. Expected number of statistically significant studies is estimated using the point estimate of the largest study (smallest standard error) as the plausible effect size

### Assessment of epidemiological credibility

Of the 113 associations, only two cardiovascular outcomes (cardiovascular mortality and venous thromboembolism) fulfilled the necessary criteria to be categorized in the strong level of evidence (Table 1). Ten (8.9%) associations were supported by highly suggestive evidence, 6 of which were on cardiometabolic outcomes. The highly suggestive associations were all-cause mortality in general population, all-cause mortality in patients with chronic kidney disease, long-term mortality in chronic obstructive pulmonary disease (COPD) patients, long-term mortality or CVD in acute coronary syndrome (ACS)/unstable coronary heart disease (CHD)/angina patients, mortality or CVD in stable coronary artery disease (CAD) patients, CHD in general population, overall survival in hepatocellular carcinoma patients, Bath Ankylosing Spondylitis Disease Activity Index-50 (BASDAI50) in ankylosing spondylitis patients, ovarian cancer in general population, and type 2 diabetes in general population. There were 16 comparisons that were categorized in the suggestive level of evidence and 67 in the weak level. Finally, 18 comparisons did not present a statistically significant association. When we excluded the case–control or cross-sectional studies, only seven comparisons were affected. Only six of those comparisons had at least 3 remaining studies in order to be re-evaluated and for all six the evidence categorization remained the same (Additional file 1: Appendix Table 2).

When we assessed the meta-analyses in either the strong or the highly suggestive evidence category, we observed that the majority of the meta-analysis papers were on moderate study quality (9 of the 11 papers) based on an AMSTAR score between 4 and 7, and only one had a score of 8. Finally, one study [41] was a pooled analysis and therefore it could not be evaluated based on the AMSTAR tool (Additional file 1: Appendix Table 3).

### CRP levels and health outcomes reported in Mendelian randomization studies

A total of 196 primary MR analyses were identified from 37 studies [79–115] covering 82 distinct phenotypes (Table 2 and Additional file 1: Appendix Tables 4, 5). The majority of associations were investigated through two-sample MR methodologies (130 out of 196; 66%). The median number of participants included in MR studies was 26 405 (range 134 to 184 305). The most frequently examined phenotypes included cardiovascular diseases (coronary heart disease and stroke) (n = 19; 9.7%), type 2 diabetes (n = 8; 4.1%), schizophrenia (n = 8; 4.1%), and body mass index (BMI) (n = 6; 3.1%). Eighty-four MR analyses (60 unique outcomes, Table 2) used instrument variants at the *CRP* gene locus, and 112 used instruments from throughout the genome The SNPs used as instruments varied vastly among studies. The four most commonly used SNPs among the 196 MR associations were rs1130864 (n = 78; 39.8% of the comparisons), rs1205 (n = 74; 37.8%), rs2794520 (n = 74; 37.8%), and rs3093077 (n = 65; 33.2%); all these variants fall within or close the CRP gene region.

**Table 2:** Health outcome and characteristics of Mendelian randomization studies. Only studies with instruments from the CRP gene are presented. One study is selected per outcome based on the largest sample size^*a*^

Reference	Phenotype	N cases	Total N	SNPs used in the GRS instrument	Level of exposure	Metric	Causal effect estimate^b^	*P*^*c*^	MR method
Wium-Andersen et al. [101, 102]	All-cause mortality	4778	78809	rs3091244, rs1130864, rs1205, rs3093077	Per doubling of CRP	OR	1.08 (0.86, 1.34)	NR	1SMR, IPD, IV regression
Prins et al. [107]	Alzheimer disease	4663	13020	rs1130864, rs3093077	Per unit of lnCRP	OR	1.26 (0.89, 1.78)	0.2	2SMR, PSD, IVW meta-analysis
Prins et al. [107]	Amyotrophic lateral sclerosis	4133	12263	rs1130864, rs1205	Per unit of lnCRP	OR	0.79 (0.60, 1.04)	0.09	2SMR, PSD, IVW meta-analysis
Wium-Andersen et al. [101, 102]	Any cancer	12343	78809	rs3091244, rs1130864, rs1205, rs3093077	Per doubling of CRP	OR	0.94 (0.81, 1.08)	NR	1SMR, IPD, IV regression
Marott et al. [96]	Atrial fibrillation	2111	46876	rs1205, rs1130864, rs3091244, rs3093077	Per doubling of CRP	OR	0.76 (0.62, 0.93)	NR	1SMR, IPD, GLSR
Prins et al. [107]	Autism	90	1566	rs1130864, rs1205, rs3093077	Per unit of lnCRP	OR	1.02 (0.97, 1.07)	0.38	2SMR, PSD, IVW meta-analysis
Prins et al. [107]	Bipolar disorder	7481	16731	rs1130864, rs1205, rs1800947, rs3093077	Per unit of lnCRP	OR	1.17 (0.97, 1.42)	0.11	2SMR, PSD, IVW meta-analysis
Allin et al. [91]	Bladder and urinary tract cancer	531	46618	rs1205, rs1130864, rs3091244, rs3093077	Per doubling of CRP	OR	0.73 (0.42, 1.25)	NR	1SMR, IPD, GLSR
Prins et al. [107]	BMI (in SD)	NA	123864	rs1130864, rs1205, rs1800947, rs3093077	Per unit of lnCRP	MD	− 0.017 (− 0.06, 0.02)	0.41	2SMR, PSD, IVW meta-analysis
Allin et al. [91]	Breast cancer	1402	46618	rs1205, rs1130864, rs3091244, rs3093077	Per doubling of CRP	OR	1.05 (0.77, 1.43)	NR	1SMR, IPD, GLSR
Prins et al. [107]	CAD	60801	184305	rs1130864, rs1205, rs1800947, rs3093077	Per unit of lnCRP	OR	1.00 (0.93, 1.07)	0.965	2SMR, PSD, IVW meta-analysis
Kivimäki et al. [86]	Carotid intima-media thickness (mm)	NA	3016	rs1130864, rs1205, rs3093077	Per doubling of CRP (mean age of 49.2)	MD	− 0.001 (− 0.025, 0.023)	NR	1SMR, IPD, IV regression
Prins et al. [107]	Celiac disease	4533	15283	rs1130864, rs1205, rs3093077	Per unit of lnCRP	OR	0.96 (0.77, 1.21)	0.75	2SMR, PSD, IVW meta-analysis
Prins et al. [107]	Chronic kidney disease	6271	74354	rs1130864, rs1205, rs1800947, rs3093077	Per unit of lnCRP	OR	1.04 (0.88, 1.22)	0.67	2SMR, PSD, IVW meta-analysis
Allin et al. [91]	Colorectal cancer	858	46618	rs1205, rs1130864, rs3091244, rs3093077	Per doubling of CRP	OR	1.10 (0.74, 1.64)	NR	1SMR, IPD, GLSR
Wium-Andersen et al. [101, 102]	COPD	3853	78809	rs3091244, rs1130864, rs1205, rs3093077	Per doubling of CRP	OR	0.87 (0.69, 1.11)	NR	1SMR, IPD, IV regression
Dahl et al. [97]	COPD hospitalization	2285	40109	rs3091244, rs1130864, rs1205, rs3093077	Per doubling of CRP	OR	0.82 (0.59, 1.13)	NR	1SMR, IPD, GLSR
Prins et al. [107]	Crohn disease	6333	21389	rs1130864, rs1205, rs1800947, rs3093077	Per unit of lnCRP	OR	0.78 (0.65, 0.94)	0.009	2SMR, PSD, IVW meta-analysis
Prins et al. [107]	Cutaneous psoriasis	1363	4880	rs1130864, rs1205, rs1800947, rs3093077	Per unit of lnCRP	OR	1.10 (0.76, 1.59)	0.62	2SMR, PSD, IVW meta-analysis
Prins et al. [107]	DBP (mmHg)	NA	69368	rs1130864, rs1205, rs1800947, rs3093077	Per unit of lnCRP	MD	0.70 (0.20, 1.19)	0.006	2SMR, PSD, IVW meta-analysis
Wium-Andersen et al. [101, 102]	Depression	1183	78809	rs3091244, rs1130864, rs1205, rs3093077	Per doubling of CRP	OR	0.79 (0.51, 1.22)	NR	1SMR, IPD, IV regression
Prins et al. [107]	eGFRcr (in mm/min/1.73 m^2^)	NA	74354	rs1130864, rs1205, rs1800947, rs3093077	Per unit of lnCRP	MD	0.004 (− 0.01, 0.02)	0.4	2SMR, PSD, IVW meta-analysis
Sunyer et al. [84]	FEF25-75% (ml)	NA	134	rs1205	Per doubling of CRP	MD	− 1283.5 (− 2792.7, 225.7)	NR	1SMR, IPD, IV regression
Bolton et al. [98]	FEV1	NA	1224	rs1800947	CG/GG compared with CC	MD	0.01 (− 0.08, 0.11)	0.82	1SMR, IPD, Genotype used as a proxy for exposure, without further estimation
Sunyer et al. [84]	FVC (ml)	NA	134	rs1205	Per doubling of CRP	MD	− 628.0 (− 1402.8, 146.8)	NR	1SMR, IPD, IV regression
Brunner et al. [85]	HbA1c (%)	NA	4678	rs1130864, rs1205, rs3093077	Per doubling of CRP (mean age of 49)	GMR	0.996 (0.981, 1.011)	NR	1SMR, IPD, IV regression
Timpson N, 2005	HDL cholesterol (mmol/L)	NA	3206	rs2794521, rs1800947, rs1130864, rs1205	Per doubling of CRP	MD	0.006 (-0.072, 0.084)	NR	1SMR, IPD, IV regression
Brunner et al. [85]	HOMA-IR	NA	3912	rs1130864, rs1205, rs3093077	Per doubling of CRP (mean age of 49)	GMR	1.035 (0.934, 1.145)	NR	1SMR, IPD, IV regression
Wium-Andersen [101, 102]	Hospitalization or death with depression	1145	76479	rs3091244, rs1130864, rs1205, rs3093077	Per doubling of CRP	OR	0.79 (0.51, 1.22)	NR	1SMR, IPD, IV regression
Davey Smith et al. [79]	Hypertension	NR	3529	rs1800947	Per doubling of CRP	OR	1.03 (0.61, 1.73)	NR	1SMR, IPD, IV regression
Prins et al. [107]	IBD (all types)	13020	47794	rs1130864, rs1205, rs3093077	Per unit of lnCRP	OR	0.97 (0.84, 1.13)	0.7	2SMR, PSD, IVW meta-analysis
Prins et al. [107]	Ischemic stroke (all types)	3548	9520	rs1130864, rs1205, rs1800947, rs3093077	Per unit of lnCRP	OR	1.19 (0.93, 1.53)	0.16	2SMR, PSD, IVW meta-analysis
Prins et al. [107]	Knee osteoarthritis	5755	24260	rs1130864, rs1205, rs1800947, rs3093077	Per unit of lnCRP	OR	0.94 (0.78, 1.13)	0.5	2SMR, PSD, IVW meta-analysis
Viikari et al. [83]	Leptin (ng/ml)	NA	1655	rs2794521, rs3091244, rs1800947, rs1130864, rs1205	Per doubling of CRP	MD	0.02 ± 0.06	0.76	1SMR, IPD, IV regression
Allin et al. [91]	Lung cancer	678	46618	rs1205, rs1130864, rs3091244, rs3093077	Per doubling of CRP	OR	1.15 (0.67, 1.98)	NR	1SMR, IPD, GLSR
Prins et al. [107]	Major depressive disorder	9240	18759	rs1130864, rs1205, rs3093077	Per unit of lnCRP	OR	0.98 (0.81, 1.18)	0.81	2SMR, PSD, IVW meta-analysis
Casas et al. [81]	Non-fatal MI	985	5216	rs1130864	TT compared with CT/CC	OR	1.01 (0.74 – 1.38)	0.95	1SMR, IPD, multivariate logistic regression
Wium-Andersen et al. [101, 102]	Not accomplishing	16001	75504	rs3091244, rs1130864, rs1205, rs3093077	Per doubling of CRP	OR	1.09 (0.96, 1.23)	NR	1SMR, IPD, IV regression
Prins et al. [107]	Parkinson disease	5333	17352	rs1130864, rs1205, rs3093077	Per unit of lnCRP	OR	1.00 (0.85, 1.17)	0.96	2SMR, PSD, IVW meta-analysis
Wium-Andersen et al. [101, 102]	Prescription antidepressant medication use	8641	76539	rs3091244, rs1130864, rs1205, rs3093077	Per doubling of CRP	OR	0.98 (0.83, 1.15)	NR	1SMR, IPD, IV regression
Allin et al. [91]	Prostate cancer	560	46618	rs1205, rs1130864, rs3091244, rs3093077	Per doubling of CRP	OR	1.02 (0.62, 1.69)	NR	1SMR, IPD, GLSR
Prins et al. [107]	Psoriasis vulgaris	4007	8941	rs1130864, rs1205, rs1800947, rs3093077	Per unit of lnCRP	OR	1.23 (0.96, 1.57)	0.11	2SMR, PSD, IVW meta-analysis
Prins et al. [107]	Psoriatic arthritis	1946	6880	rs1130864, rs1205, rs1800947, rs3093077	Per unit of lnCRP	OR	1.45 (1.04, 2.04)	0.03	2SMR, PSD, IVW meta-analysis
Davey Smith et al. [79]	Pulse pressure (mm Hg)	NA	3529	rs1800947	Per doubling of CRP	MD	− 0.40 (− 5.38, 4.57)	NR	1SMR, IPD, IV regression
Prins et al. [107]	Rheumatoid arthritis	5538	25702	rs1130864, rs1205, rs1800947, rs3093077	Per unit of lnCRP	OR	0.94 (0.77, 1.15)	0.55	2SMR, PSD, IVW meta-analysis
Prins et al. [107]	SBP (mmHg)	NA	69372	rs1130864, rs1205, rs1800947, rs3093077	Per unit of lnCRP	MD	1.23 (0.45, 2.01)	0.002	2SMR, PSD, IVW meta-analysis
Hartwig et al. [109]	Schizophrenia	35476	82315	rs1130864, rs1205, rs1800947, rs3093077	Per 2-fold of lnCRP	OR	0.93 (0.86, 1.00)	0.04	2SMR, PSD, weighted generalized linear regression
Wium-Andersen et al. [101, 102]	Self-reported antidepressants	5002	75169	rs3091244, rs1130864, rs1205, rs3093077	Per doubling of CRP	OR	1.16 (0.95, 1.43)	NR	1SMR, IPD, IV regression
Prins et al. [107]	Serum albumin level (gr/dl)	NA	53189	rs1130864, rs1205, rs1800947, rs3093077	Per unit of lnCRP	MD	− 0.002 (− 0.02, 0.01)	0.77	2SMR, PSD, IVW meta-analysis
Prins et al. [107]	Serum protein level (gr/dl)	NA	25537	rs1130864, rs1205, rs1800947, rs3093077	Per unit of lnCRP	MD	0.008 (− 0.02, 0.04)	0.64	2SMR, PSD, IVW meta-analysis
Prins et al. [107]	Systemic lupus erythematous	1311	4651	rs1130864, rs1205, rs3093077	Per unit of lnCRP	OR	1.20 (0.80, 1.81)	0.38	2SMR, PSD, IVW meta-analysis
Prins et al. [107]	Systemic sclerosis	2356	7518	rs1130864, rs1205, rs3093077	Per unit of lnCRP	OR	1.07 (0.78, 1.45)	0.68	2SMR, PSD, IVW meta-analysis
Rode et al. [104]	Telomere length in base pairs	NA	45069	rs3091244	Per doubling of CRP	MD	− 66 (− 124, − 7)	NR	1SMR, IPD, IV regression
Timpson et al. [80]	Triglycerides (mmol/L)	NA	3206	rs2794521, rs1800947, rs1130864, rs1205	Per doubling of CRP	GMR	0.99 (0.92, 1.08)	NR	1SMR, IPD, IV regression
Prins et al. [107]	Type 1 diabetes	9934	26890	rs1130864, rs1205	Per unit of lnCRP	OR	1.15 (0.90, 1.47)	0.26	2SMR, PSD, IVW meta-analysis
Prins et al. [107]	Type 2 diabetes	6698	22570	rs1130864, rs1205, rs1800947, rs3093077	Per unit of lnCRP	OR	1.11 (0.94, 1.32)	0.23	2SMR, PSD, IVW meta-analysis
Prins et al. [107]	Ulcerative colitis	6687	26405	rs1130864, rs1205, rs1800947, rs3093077	Per unit of lnCRP	OR	1.10 (0.92, 1.31)	0.29	2SMR, PSD, IVW meta-analysis
Zacho et al. [92, 93]	Venous Thromboembolism	1370	46470	rs3091244, rs1130864, rs1205, rs3093077	Per doubling of CRP	OR	0.80 (0.56, 1.12)	NR	1SMR, IPD, GLSR
Timpson et al. [80]	Waist-to-hip ratio	NA	3206	rs2794521, rs1800947, rs1130864, rs1205	Per doubling of CRP	MD	0.005 (− 0.007, 0.016)	NR	1SMR, IPD, IV regression
Wium-Andersen et al. [101, 102]	Wanting to give up	4846	75694	rs3091244, rs1130864, rs1205, rs3093077	Per doubling of CRP	OR	1.02 (0.83, 1.26)	NR	1SMR, IPD, IV regression

*1SMR* one-sample Mendelian randomization; *2SMR* two-sample Mendelian randomization; *BMI* body mass index; *CAD* Coronary artery disease; *COPD* chronic obstructive pulmonary disease; *CRP* c-reactive protein; *DBP* diastolic blood pressure; *FEF* forced expiratory flow; *FEV1* Forced expiratory volume in 1 s; *FVC* Forced vital capacity; *GLSR* Generalized least squares regression; *GMR* Geometric Means Ratio; *HDL* high density lipoprotein; *HOMA*-*IR* Homeostatic Model Assessment for Insulin Resistance; *HR* Hazard ratio; *IBD* irritable bowel syndrome; *IPD* individual participant data; *IV* Instrumental variable; *IVW* Inverse variance weighted; *MD* mean difference; *MI* Myocardial infarction; *NR* not reported; *OR* odds ratio; *PSD* published summary data; *RR* Relative risk; *SBP* systolic blood pressure; *SNP* single nucleotide polymorphism

^a^Full list of Mendelian randomization studies in Additional file

^b^Causal effect estimate of all variants combined

^c^Causal effect *P*-value

Overall, 12 distinct phenotypes presented significant associations at a *P *< 0.01, of which four (Crohn’s disease, ischemic heart disease, systolic and diastolic blood pressure) presented significant associations (*P *< 0.01) when the instruments were restricted to CRP gene locus (Appendix Tables 4 and 5). However, independent MR analyses did not show consistent evidence for Crohn’s disease and ischemic heart disease, and none of the aforementioned phenotypes had support from sensitivity analyses.

Nine phenotypes presented significant (*P *< 0.01) causal effect estimates when instruments from throughout the genome were considered and of those, only schizophrenia and bipolar disorder presented consistent evidence in sensitivity analyses and in analysis restricted to SNPs within CRP locus, but only at *P *< 0.05. Nonetheless, the result on bipolar disorder [113] was not confirmed by an earlier study [107] where MR using only CRP gene SNPs did not reach statistical significance at *P *< 0.05. Schizophrenia had evidence from independent studies and sensitivity analysis (weighted median and inverse variant weighted estimate), but this was not supported by MR Egger analysis and the sensitivity analysis using only CRP gene SNPs (*P *= 0.04).

Overall, only 14 outcomes had evidence available from both MR analyses and meta-analyses of observational studies (Table 3). The evidence between the observational studies and MR analyses was concordant for three outcomes where both meta-analyses of observational studies and MR analyses were not statistically significant (*P *≥ 0.05). The remaining studies showed various degree of evidence (weak, suggestive, highly suggestive) with meta-analyses of observational studies and no evidence or limited inconsistent evidence from MR. Finally, MR did not support causality for venous thromboembolism whose evidence was graded as strong in the observational meta-analysis evidence.

**Table 3:** Comparison of evidence from observational studies meta-analysis and Mendelian randomization (MR) studies taking into account both CRP gene-only and genome-wide significant instruments

Population (observational)	Outcome (observational)	Grade (observational)	Outcome (MR)	Grade (MR)
General population	Venous thromboembolism	Strong	Venous Thromboembolism	No evidence
General population	All-cause mortality	Highly suggestive	All-cause mortality	No evidence
General population	Coronary Heart Disease	Highly suggestive	Coronary Heart Disease	No evidence
General population	Type 2 diabetes	Highly suggestive	Type 2 diabetes	Limited/inconsistent evidence
General population	Hypertension	Suggestive	Hypertension	No evidence
General population	Ischemic stroke	Suggestive	Ischemic stroke (all types)	No evidence
AF patients	Atrial fibrillation (recurrence)	Weak	Atrial fibrillation	No evidence
General population	Alzheimer’s disease	Weak	Alzheimer’s disease	Limited/inconsistent evidence
General population (women)	Breast cancer	Weak	Breast cancer	No evidence
General population	Colon cancer	Weak	Colon cancer	No evidence
General population	Colorectal cancer	Weak	Colorectal cancer	Limited/inconsistent evidence
Vascular surgery patients	Non-fatal Myocardial Infarction	No evidence	Non-fatal Myocardial Infarction	Limited/inconsistent evidence
General population (men)	Prostate cancer	No evidence	Prostate cancer	No evidence
General population	Rectal cancer	No evidence	Rectal cancer	No evidence

## Conclusions

Our umbrella review showed an impressive body of literature on CRP including 113 comparisons from 55 studies for separate phenotypes and 196 MR analyses to assess causality of epidemiologic associations. Only 14 phenotypes had evidence from meta-analysis of observational studies and MR analyses. Most summary meta-analytic estimates of observational studies yielded nominally statistically significant results for a direct association between CRP and different phenotypes. Nonetheless, only two of these associations had strong results with no suggestions of biases (cardiovascular mortality and venous thromboembolism in general population) and none of these had supporting evidence of a causal role for CRP in MR investigations.

Low-grade inflammation has been suggested to be involved in many chronic diseases, which may explain the breadth and depth of phenotypes examined in relation to CRP, a general marker of inflammation that can be inexpensively measured in epidemiological and clinical settings. A search of “C-reactive protein or CRP” yields 74,622 items as of March 05, 2019, and the vast number of meta-analyses that we identified are efforts to summarize this huge, expanding literature.

A large proportion of studies examined CRP as a prognostic marker of cancer incidence but also of cancer survival. Out of those 52 comparisons, there was highly suggestive evidence for only two associations (ovarian cancer incidence and overall survival in hepatocellular carcinoma). The evidence from the remaining literature was classified as suggestive or weak. MR efforts, including one on lung cancer, did not highlight any evidence of causality either, although their sample sizes were modest for less common cancers. Chronic inflammation may still be linked to cancer development and progression, as other lines of evidence suggest a higher risk of cancer amongst individuals with inflammatory conditions (e.g., inflammatory bowel diseases and risk of colon cancer), or higher risk of cancer in relation to infections (e.g. human papillomaviruses and cervix cancer) [115–119]. However, CRP, as a general marker of inflammation, is unlikely to capture the specific inflammatory mediating pathways linking inflammation to cancer development and progression.

CRP and cardiovascular diseases have been subject to an increasing body of research and debate. Our review found that the associations of CRP with cardiovascular mortality and venous thromboembolism were supported by strong evidence. Furthermore, we found highly suggestive evidence between higher CRP and risk of CHD, type 2 diabetes and mortality or CVD on stable CAD patients and on unstable CHD/ACS/angina patients. Nonetheless, MR studies have repeatedly failed to provide evidence for causal association with CHD; an observation further supported from randomized controlled trials [120]. The observational literature of CRP is likely to suffer from diverse biases and the effect size of the associations may be inflated [121, 122]. Beyond causality, even efforts to show that CRP could at least be used in risk prediction have also not demonstrated convincing results [123, 124]. Accordingly, the relative risks that we noted for cardiovascular mortality (2.05, in fact just 1.49 in the largest study) and venous thromboembolism (only 1.14) do not suggest a substantial predictive potential. The role of inflammation in atherosclerotic plaque initiation, progression and rupture has been supported by various other lines of evidence [125], but this may not necessarily prove that CRP should have clinical utility.

COPD is associated with an abnormal inflammatory response beyond the lungs with evidence of low-grade systemic inflammation which causes systemic manifestations such as weight loss, skeletal muscle dysfunction, an increased risk of cardiovascular disease, osteoporosis and depression [125–128]. We found highly suggestive evidence that CRP is associated with late (but not with early) mortality in COPD patients. However, MR studies did not support a causal association. CRP might be elevated in COPD patients due to reverse causality as the disease is associated with triggering an inflammatory response. Reverse causality is likely to explain other associations such as mortality in patients with chronic kidney disease or overall survival in hepatocellular carcinoma patients. In these instances CRP could serve as a predictive factor for disease severity, but studies assessing its value over and above validated existing risk prediction algorithms are essential to support any prediction claim [123].

Some particular mention needs to be made on schizophrenia, where, among the tentative MR findings described in this review we found several studies of CRP and schizophrenia onset. Yet, there is a distinctive lack of observational data on this association, and those that exist [129, 130], mainly focus on the reverse pathway of the association (how schizophrenia affects CPR levels) than what is the focus of this review.

In our MR review we found multiple studies and sensitivity analyses show evidence for causal effect, but with very modest *P*-values, when only CPR SNPs were used in the genetic instruments. One recent analysis (published after the search date of our review [131]) found even lower *P*-values with inverse variance weights and generalized summary MR modeling. The putative causal association with schizophrenia is even more interesting because it suggests a protective effect of CRP on schizophrenia, while observational data had suggested an association of CRP with higher schizophrenia risk [130].

Overall, the overwhelming majority of the meta-analyses of observational studies reported a nominally statistically significant result (84%) in contrast to MR studies where only 37 of the 196 (19%) analyses presented nominally statistically significant results. These two study designs may be subject to different biases in the biomedical field. A large proportion (48.2%) of the examined observational meta-analyses displayed substantial heterogeneity (I^2^ > 50%), small study effects (39.5%), and excess significance bias (41.2%), which, in addition to the small effect estimates increase the probability of false-positive findings. MR approaches use genetic variants as instrumental variables to establish whether an exposure is causally related to a disease or trait. The genetic variants are unrelated to confounding factors, and therefore, this approach is not as prone to confounding and reverse causation bias. At the same time, genetic association estimates in MR represent the average lifetime association of the variants with the outcome for all those in the considered population, and are therefore less vulnerable to measurement error [132]. Nonetheless, MR also shares some of the limitations of observational epidemiology literature including small sample sizes, instrument bias and low power, and poor reporting has further additional limitations [22]. For example, we observed that at least half of the MR studies on CRP used instruments derived from genome-wide association studies including genetic variants on genes of other inflammatory cytokines such as IL-6. These approaches may introduce potential pleiotropy and can thus bias MR estimates. There are several methodologies to account for the violation of the pleiotropy assumption of MR, but these cannot always identify pleiotropic effects, and therefore, can only partly disentangle the complex pleiotropy previously shown between CRP and lipid and metabolic pathways [133].

Limitations of our approach need to be acknowledged. Our review focused on existing meta-analyses, and therefore, outcomes that were not assessed in a meta-analysis are not included in this review. Furthermore, we did not appraise the quality of the individual studies but the quality of the actual meta-analyses. We refer interested readers to the quality assessments already made by the authors of each original meta-analysis and we did not wish to change the eligibility criteria based on quality since this would add our own subjective in study selection. We did not include evidence from randomised control trial meta-analyses as these examine a wide range of anti-inflammatory treatments which are not specific to CRP lowering effects. Statistical tests for small-study effects and excess significance bias should also be interpreted with caution in case of large between-study heterogeneity and both tests have limited power in the presence of few studies or sparse studies with significant results. Finally, we adopted credibility assessment criteria, which were based on established tools for observational evidence; however, none of the components of these criteria provides firm proof of credibility of evidence, but they cumulatively describe the possibility that the results are susceptible to bias and uncertainty.

In this extensive systematic review of meta-analyses of observational studies on CRP and disease outcomes and of the evidence stemming from MR studies, we could not find strong evidence supported by both study designs in relation to CRP and the most frequently studied non-infection phenotypes in the literature. Observational studies presented robust evidence of association between higher CRP levels and cardiovascular mortality and venous thromboembolism, but without causality support from MR studies. Following claims that CRP maybe be a novel CVD risk factor [134], it has been extensively studied in relation to an ever-increasing list of phenotypes and diseases, but it does not seem to be crucially relevant to any of them. Despite intensive research efforts, our study shows that there is little evidence that CRP may constitute a priority interventional target for any diseases.

## Electronic supplementary material

Below is the link to the electronic supplementary material.

Supplementary material 1 (XLSX 75 kb)
